# Regime Change: Education to Work Transitions in England, 1980s–2020s

**DOI:** 10.1007/s43151-020-00004-6

**Published:** 2020-06-25

**Authors:** Ken Roberts

**Affiliations:** Sociology Department, University of Liverpool, Eleanor Rathbone Building, Bedford Street South, Liverpool, L69 7ZA England

**Keywords:** Education, Labour markets, Transition regime, Vocational training, Youth

## Abstract

Abstract

This paper argues the case for viewing a country’s transition regime as a totality in which different career steps and routes assign significance and value to one another. Following the destruction of major sections of Britain’s transition regime in the 1970s and 1980s, the paper explains how the regime has subsequently been reconstructed following the 1988 Education Reform Act, replacing the vocational education and training schemes of the 1980s with government-supported apprenticeships, almost uninterrupted employment growth since 1992, and the financialization of the corporate economy alongside uncapped expansion of higher education. England’s current transition regime keeps rates of youth unemployment and NEET below the European Union averages and delivers the fastest transitions in Europe. However, the reconstructed regime also locks-in features that may well become long-term problems, namely an expensive higher education system which leaves graduates with debts that many do not expect to repay in full, and substantial low wage, low skill, low productivity sectors in the economy and workforce.

## Introduction

### Aims

In retrospect, we can understand the 1970s and 1980s as decades when major sections of Britain’s former education-to-work regime were destroyed by globalization, deindustrialisation and neo-liberal economic policies. A series of efforts were made to shore-up old and to build new bridges into work. These included a Technical and Vocational Education Initiative and new employer-led vocational qualifications (BTECS and NVQs). There were wage subsidies subject to rates of pay beneath a low ceiling offered by a Young Workers Scheme followed by a New Workers Scheme which were intended to depress youth pay to a level at which unemployment would clear. A Job Release Scheme permitted early retirement on full pensions provided the vacated job was filled by an otherwise unemployed young person. A Youth Opportunities Programme was superseded by successive versions of a Youth Training Scheme, first launched in 1983, relaunched in 1986, then for a third and final time in 1990 rebranded as simply Youth Training. There was support for young people to start their own businesses. Nothing worked! Some young working lives recovered during the following decades, but others suffered long-term from their early scarring experiences (Droy et al. 2019; Williamson 2004), The first of the incoming Labour government’s ‘new deals’ in 1997 was for 18–25 year olds who had been failed by earlier measures. These, and the replacement of the Careers Service with Connexions which was given the priority aim of reducing the numbers of young people who were not in employment, education, or training (NEET), were the final attempts to restore old routes into employment.

Again in retrospect, the last 30 years can be seen as a period of reconstruction, creating a youth transition regime adapted to post-industrial Britain in a global, neo-liberal age. The aim of this paper is to map the reconstructed regime with which England has entered the 2020s and to explain how it has been built.

### The Story

Prior to the 1980s, young people entered the former transition regime at age 16 having been divided into two main groups. These were a minority with grammar school qualifications and the rest. There were three main groups of occupational destinations, employment classes, to which young people could head: a middle class with careers in management and professional occupations, an intermediate class of office staff and technicians, and a working class. Two start points and three possible destinations meant that six journeys were possible, but only some of these possible journeys became actual career routes and groups. This depended on which young people employers would recruit, those who intermediary education and training providers would admit, and the preferences and choices of young people from the opportunities that were open to them.

Young people today are divided into two groups when they enter the changed regime, now at age 18. The groups are different from their pre-1980s counterparts. They are now the majority who have the qualifications for higher education and the rest. There are now four employment classes to which young people can head: an upper middle class that is entered through careers into elite management and professional occupations, a lower middle class who are employed in offices and laboratories, a new upper working class, and a precariat. Thus there are now eight possible journeys, but only five are activated by the interactive agency of employers, intermediaries, and young people. The activated routes are as follows:

i. University into elite careers
ii. University into the lower middle class
iii. Exit education at 18 and enter the lower middle class, possibly via an apprenticeship
iv. Exit education at 18 and enter the upper working class, possibly via an apprenticeship
v. Fail in education and join the precariat

The following passages explain how the two groups of young people at the point of entry, the four employment class destinations, and five main activated routes have been formed since the 1980s by four change drivers. These are as follows:

i. The 1988 Education Reform Act, which is still the most recent in England’s major education acts (its predecessors were in 1870, 1902 and 1944)
ii. The replacement of the 1980s Youth Training Schemes and other measures with the present-day apprenticeship programme
iii. The growth in employment which began in 1992 when Britain exited the Exchange Rate Mechanism (the precursor of the hard Euro), stalled only briefly during the financial crisis of 2007–2009, and has continued since 2010 in apparent defiance of government austerity policies resulting in 2019 in record levels and rates of employment, and the lowest unemployment rate since the 1970s
iv. The financialization of the UK corporate economy which has spread following the deregulation of finance businesses in the 1980s, and has created elite careers leading into an upper middle class which has pulled well ahead of other management and professional (service class) occupations

However, we must proceed by explaining the key concepts that feature throughout the analysis. These concepts are youth, transitions, regime, and career routes/pathways/trajectories (forthwith ‘routes’ will be the term used).

### Youth Transitions

Over 50 years ago when psychology was still the lead discipline in youth research, the terms ‘youth’ and ‘adolescence’ were often used interchangeably. Youth was an age group, and delinquency and provocative youth styles in dress and tastes in music were explained as products of the bio-psycho turmoil that followed puberty.

All this changed when the youth life stage began to lengthen and was decoupled from adolescence. Young people’s emancipation from family controls in choices of friends, dress, and music was commencing earlier, and the life stage was stretching upwards as young people remained longer in education, and ages rose when individuals began continuous full-time employment careers, married, and became parents. Sociologists (e.g. see Furlong 1992; Wallace and Kovacheva 1998) insisted that the life stage was a social construct and became the lead scholars in youth studies. Start and end ages of the life stage were shown to vary by time and place and between socio-demographic groups. By the 1980s, there was a sociological consensus that youth was not only lasting longer but also involved more transitions, that is, more switches between courses in education, training, and jobs as well as in housing and intimate relationships (see Martin-Garcia et al. 2017). The new orthodoxy in sociology was that youth life stage transitions were becoming more complex and not necessarily linear with more reverses back into parents’ homes and from the labour market back to education, although a linear transition still appears to remain normative (e.g. see Aeby and Heath 2019). From the 1980s onwards, the youth research field could be described as the study of youth life stage transitions, not an age group, and the task of research was to plot how journeys through the life stage varied not just by time and place, but also by family class and composition, education, gender, ethnicity, sexuality, abilities and disabilities, etc. By the 1980s the post-war decades of full employment had ended, youth unemployment was becoming an issue from country-to-country throughout Europe, and the education-to-work transition replaced delinquency and youth cultures as the main topic addressed by youth researchers.

### Transition Regimes

This concept could also be used in studies of young people’s housing, but in practice, its use has been confined to research on education-to-work transitions, usually in multi-country comparative projects. With hindsight, this now appears to have been a methodological error.

One sustained initiative has been by the European Research Network on Transitions in Youth (TiY) which has met annually since its inaugural event in 1993. The core members of the original network had conducted quantitative studies in their respective countries of young people’s routes directly from lower secondary education into employment or via upper secondary education or various forms of vocational education and training (e.g. see Brauns et al. 1997; Shavit and Muller 1998). These researchers were unable to harmonize their data sets. There were no direct equivalents to the types of schools, qualifications, and training that specific countries had developed. Therefore, the network decided that multi- country comparisons would be possible only if they could identify a limited number of transition regime types into which each country could be slotted (see Raffe 2008). This aim has not yet been achieved, and the TiY network’s quest for a comprehensive typology has stalled (Raffe 2014). Hence, education-to-work comparative researchers have sometimes resorted to Esping-Andersen’s typology of welfare regimes (e.g. Hammer 2003; Pohl and Walter 2007) but have found that inter-country differences in education, training, and employment do not map neatly onto the welfare regime types.

The sole ‘types’ that have been used extensively in comparative education-to-work research have distinguished regulated or occupationalized from unregulated or non-occupationalized regimes (Gangl 2001; Szydlik 2002). However, these are not discrete categories but poles of a continuum, usually operationalized in research according to the relative proportions of the upper secondary age group who follow academic or general courses on the one side, and vocational education and training on the other (e.g. see Hadjivassiliou Kari et al. 2019; Kogan et al. 2011; Kogan and Unt 2008; Lange et al. 2014; Noelke et al. 2012). The problem here is that the academic/general ‘type’ of education may simply be non-specific vocationally or deemed more intellectually demanding than the alternatives as in the traditional English grammar school, the German gymnasium, and the classical French lycee. At the other end of the continuum, vocational education and training differ in how securely they are linked to employment destinations and the levels of these occupations in the countries’ class structures. Scandurra et al. (2020) have used Pohl and Walter’s (2007) typology which enhances Esping-Anderson’s types of welfare regimes with information on typical mixes of academic/general and vocationally specific education and training, to analyse EUROSTAT data on relationships between levels of educational attainment, NEET, and youth unemployment rates from 2005 to 2016 across the whole of Europe. They find differences between regime types, but also differences by country within regime types plus, in some countries, substantial regional differences.

A second sustained initiative on transition regimes has been by researchers originally self- described as the European Group for Integrated Youth Research (EGRIS) (2001). The members of this group have conducted comparative qualitative studies of selected groups of young people in their respective countries. Their approach is said to be ‘integrated’ in exploring all aspects of young people’s lives. The focus has consistently been on the education-to-work transition which is then shown to be influenced by and to affect family relationships and housing circumstances. This research has highlighted young people’s agency, stressing how its uses always depend on the opportunities that are available in local areas, the support that families are able and willing to offer, state welfare support, and advisory services, always within a country’s distinctive transition regime (Eichhorst et al. 2015; Pohl and Walter 2007; Walther 2006, 2012, 2015, 2017).

This paper uses ‘transition regime’ differently, as recommended by Raffe in 2014, namely, in a case study approach which, in this case, means treating England’s education, training and employment, and the links between them as a ‘total’ regime and seeks to explain the construction of the main routes through the regime that individuals can follow. A ‘total’ approach is essential because the various parts of any regime do not just interact but assign meaning to each other. For instance, an English 16–18 year old studying GCE A-levels signals that the student performed adequately in the 16-plus GCSE examinations and is progressing along a route with open gates to higher education. Each step in a transition regime derives its significance from the typically preceding and following steps. Every series of steps (career routes) acquires meaning only when the typical origins and destinations of its participants are compared with those on parallel routes.

In politically liberal market economies, governments cannot impose transition regimes in total and in detail. The types of education that governments will fund and permit, and likewise the training provisions and welfare support for young people, together with employment regulations, set the context in which educational actors, employers, training providers, and young people pursue their own agendas. The detailed links between steps and the boundaries between routes are negotiated by the interactive agency of all these parties. Thus the links and boundaries change over time as they are constantly renegotiated in local education and labour markets, but always within recognizably national transition regimes. A regime is surrounded by an economy that creates the classed family backgrounds from which young people approach the transition regime and the employment classes that are their possible destinations.

### Career Routes and Groups

This paper is not the first attempt to identify young people’s main routes into the workforce in twenty-first century England. In the early-1990s, when the European Research Network on Transitions in Youth was formed, the researchers had their own countries’ data sets that had been gathered for similar purposes and in similar contexts of lengthening youth transitions. In England there was the Youth Cohort series of follow-up studies of 16 year olds that had commenced in 1985 (see, e.g. Courtenay 1988). Scotland had a series of school-leaver surveys that had begun in the 1970s and over time had become more longitudinal (see Furlong 1992; Furlong and Raffe 1988; Taylor 1996). More recent investigators have been able to use larger, even more longitudinal, data sets including those from the birth cohort studies and the British Household Panel Survey which commenced in 1991 and has subsequently been absorbed into the even larger Understanding Society project.

Schoon and Lyons-Amos (2016) have used the latter data set to compare the pathways from age 16 of cohorts born in 1980–1984 and 1985–1989. They use a combination of sequence and cluster analysis which shows that 88% of the older cohort and 78% of the younger cohort had followed one of three pathways as follows: leave education at 16 then continuous employment, stay-on but not into higher education then continuous employment, and higher education. The main inter-cohort change had been a decline from 30% to 18% in those leaving education at 16 and starting employment immediately and in a much smaller fourth pathway which was marked by repeated episodes of unemployment. The numbers on this pathway rose from 5 % to 16% between the older and younger cohorts. The authors explain this change in terms of a deterioration in the supply of jobs following the 2008 financial crisis, but an alternative explanation is a longer-term decline in employment for 16 year olds in manufacturing and extractive industries, and a partly compensating growth in precarious employment that began in the 1990s. The proportions of the cohorts who were simply ‘inactive’ (in education and the labour market) after age 16 changed minimally between the cohorts at six and 7 %.

In the similar analysis of a similar data set from a nationally representative Australian panel study, Ranasinghe et al. (2019) found that 83% of the sample had followed the same three pathways that accounted for 88 and 78% of Schoon and Lyons-Amos’s two British cohorts. Even more remarkably, these three pathways bear a close resemblance to those identified by Ashton and Field (1976) in their analysis of the findings from a qualitative survey of school-leavers in Leicester in the early-1960s. One group had embarked on ‘long careers’ launched in extended education, a second group had short careers which included apprentices who trained for skilled occupations, and a group with ‘no careers’ had moved directly into non-skilled work at age 15 or 16. The Schoon and Lyons-Amos findings suggest that over the next decades nothing changed except the proportions following their three main pathways, which seems remarkable given that the youth life stage is supposed to have lengthened and transitions become more complicated.

### Methods Fit-for-Purpose

The Schoon and Lyons-Amos findings are technically correct but cannot be the whole truth. A major limitation of sequence analysis is its difficulty in handling simultaneously differences in the education received by individuals in the same age group and between different classes of employment. Introducing these distinctions creates an incomprehensible number of different sequences which is resolved by simplification into any education or any employment.

Moreover, pathways that are identified solely with data from young people about their own life courses produces a relentless focus on young people’s agency. A total transition regime approach identifies additional agents—education and training providers and employers—and ultimately it is employers who decide which young people will be admitted to different classes of employment. There is a massive disconnect between the career aspirations of England’s young people which on aggregate appear to remain stable from ages seven and eight to 17 and 18 and the jobs that they can enter (Chambers et al. 2020). The coherence with which 20-somethings describe their biographies, and their claims to be responsible and in control of their lives, are constructed retrospectively (Devadason 2007). Young people do not become trapped in precarious employment by a poverty of aspiration. As explained below, ‘underachievement’ in education, failure at its most extreme, is socially constructed, and it is employers, not young people, who create precarious jobs.

Mapping an entire transition regime needs to draw on multiple sources of evidence. These include longitudinal surveys of young people but there is also much to learn from cross-sectional studies, in both cases from nationally representative samples but also from selected groups. Government administrative data is a valuable resource. The subjects who are investigated must be not just young people but all the actors—educators, trainers, and employers—who construct, maintain, and change transition regimes. The sources of evidence in the mapping that follows are cited when they contribute.

Examining change over a 30-year period, which is how long reconstruction in England has taken, enables us to identify not just young people’s main routes as they exist now, but how these routes have been formed, and to identify the drivers of change. Government policies and actions are invariably involved, but regime reconstruction has depended on how educators, trainers, employers, and young people have responded to these government policies and actions. The following passages claim that the main change drivers in England have been fourfold.

We shall see that the manner in which the various parties have responded following the 1988 Education Act has created just two groups at the point of entry to the transition regime while economic changes have created four clusters of occupations (employment classes) into which young people can exit. Meanders are possible and frequent but there are just eight possible journeys between ports of entry and destinations. One journey looks impossible while another two appear improbable. Thus, there are five main pathways, all with digressions, that young people can follow. Needless to say, this is not how their prospects and opportunities will appear to any of the actors in the transition regime who all view the education, training, and employment landscapes from their own specific vantage points.

## Regime Reconstruction

### The Former Transition Regime

Since the topic is change, it is necessary to start with the transition regime that has been replaced, and to see this regime in action, we need to look further back beyond the 1980s by when swaths of the old regime had disappeared or were crumbling, and 1972 is a convenient start point because this is when the school-leaving age was raised from 15 to 16 and employers worried that they would be denied a year’s intake of school-leavers.

At that time, young people were divided into the career groups described by Ashton and Field (1976). Seventy per cent (but declining) left full-time education at the earliest opportunity and nearly all stepped directly into jobs. This was the era of swift, one step school-to-work transitions. Anyone with grammar school qualifications (GCE O- and A-levels), and even those with passes in the less prestigious Certificate of Secondary Education (CSE) that had been launched in 1965, could access at least a short career into, then rising up the office grades, or via an apprenticeship into a skilled working class job. Over 10% were progressing into higher education. This figure had risen from 3 % in the late-1940s and would continue to rise. Graduates had no difficulty to accessing long careers in management and the professions. However, most recruits to these occupations followed the ‘alternative route’, leaving school with GCE passes, or less, then adding to these in further education (see Roberts 2020). Young people who left school with no qualifications usually entered non-skilled working class jobs in which they could advance rapidly to full adult earnings. The gap between skilled and non-skilled wages had been narrowing since the 1940s, so Willis’s lads were able to celebrate having exposed the idiocy of teachers who had stressed the value of qualifications (Willis 1977).

The employment classes into which young people exited the transition regime were those identified in the class scheme developed by Goldthorpe for initial use in his 1972 study of social mobility (Goldthorpe et al. 1987). There was a service class of managers and professions, intermediate classes which included low level office staff, and working classes. Unqualified 16 year olds would enter working class occupations. Higher education graduates would enter the service class. Young people who quit full time education at 16–18 with grammar school qualifications could enter the lower middle class of office staff, or the working class (usually the skilled occupations), with the possibility of further ascent using the ‘alternative route’ on which attrition rates were much higher than in higher education.

Thus, there were two start points from which young people entered the transition regime in the early 1970s. The divide was between those with and without grammar school qualifications (which were as likely to be earned in a comprehensive as in a grammar school by the 1970s). There were three employment classes into which they could exit the transition regime. Entry and exit points were linked by four main routes. Young people with grammar school qualifications at 16 could progress via A-levels and higher education into the service class, or they could exit education at 16–18 into lower middle class or (invariably skilled) working class jobs, with prospects of further progression into the service class. Sixteen year olds with inferior or no qualifications would enter (usually non-skilled) working class jobs.

During the 1970s, globalization and de-industrialization began to destroy the routes that less qualified school-leavers had followed. This destruction continued throughout the 1980s. The impact can be seen among the 1958 birth cohort. These young people became eligible to leave education in 1974 when economic restructuring was beginning. By age 33, 42% had experienced one or more spells of unemployment (Makepeace et al. 2003). Comparable data was not collected from the 1946 birth cohort. Unemployment had simply not been an issue when they entered the transition regime. However, non-manual employment continued to expand throughout the 1970s and 1980s, especially in the management and professional grades. This was a long-term trend: the jobs accounted for around 20% of employment in the 1940s, rose to around 40% by 1990, then stabilized (Goos and Manning 2003). This was the context in which, throughout the 1970s and 80s, more young people stayed longer in education, achieving better qualifications than their predecessors. The proportion entering higher education had risen to 30% by the early-1990s (Mason 1995). Their career routes were not part of the destruction. Attempts to shore-up routes that were crumbling continued throughout the 1990s but by then a new education act had been passed and, although unknown at that time, processes of reconstruction had started.

### Compulsory Education and the 1988 Education Reform Act

Since 2015, young people have been legally required to remain in full-time learning until age 18. By then, they are polarized. Around two-thirds have the qualifications required to enter higher education. They do not all take this route immediately, but they can all enter if they wish. Since 2015 when the government lifted the cap on the numbers that each university could admit, there has been an excess of places. Some course, at some university, will admit any student with its basic entry qualifications.

The remaining third fail to obtain the grades in the 16-plus GCSE examination that would allow them to progress to courses that qualify for entry to higher education (Department for Education 2019; OFQUAL 2019). Few improve their educational attainments after age 16 (Augar Repor 2019; Wolf 2011). They do not all enrol at a school or college. Labour Force Surveys show that at any point in time, around 15% of 16 and 17 year olds are not in full-time education (see Table 2, below). Those who are enrolled do not all take an examination. If they do so, those who ‘fail’ at age 16 rarely improve on their results subsequently. According to the Children’s Commissioner (2019), in 2018 18% of England’s 19 year olds left education with ‘nothing’, meaning that they had still not gained the qualifications which would admit them to subsequent courses that could lead to higher education. Around a fifth of England’s young people leave education without the levels of literacy and numeracy that are needed to function normally in everyday life (Brooks et al. 2001). England is close to the top of two European ‘league tables’: in the percentage of young people who qualify for and eventually enter higher education, and the percentage who leave education lacking basic skills. This is a remarkable outcome. It is not a statistically normal distribution. It is a product over time of the 1988 Education Reform Act (see Tomlinson 2005).

The 1988 Act removed from local authorities the ability to coordinate and encourage cooperation between the schools in their areas. Schools became independent units for assessing their work and distributing funds. The Act aimed to raise standards of attainment and its chosen method was competition. It introduced a national curriculum and national tests of pupils’ attainments at ages seven, 11, and 14 (these tests were soon discarded), and in a GCSE 16-plus examination which would be taken by all pupils. An intended product has been league tables which compare the performances of schools in specific areas. These league tables were intended to influence parents’ choices of schools for their children, and this has indeed been an outcome. High performing schools attract increased applications and may be able to expand. Pupil numbers drive school budgets up or down. Schools at the bottom of local league tables can expect pupil numbers to decline which means reduced funding, fewer teachers, and a spiral of descent which leads to more visits from school inspectors and, very likely, eventual closure.

Under these pressures, primary and secondary schools strive to gain the best possible results. Setting by ability in secondary schools is standard practice. The most able pupils will be taught and pushed to gain the highest grades that are available in all subjects. Other sets will be taught with the aim of at least a basic pass in all subjects for all pupils, and higher grades in the subjects in which different groups of students excel (Playford and Gayle 2016). Other pupils know from age 13/14 that they are expected to ‘fail’ with the predictable implications for academic confidence and effort. Hence, the polarization has widened by age 18 (see Ainley 2016).

As under the former transition regime, young people are split into two groups at the point of entry. Whereas in the former regime the division was between a minority with grammar school qualifications and the rest, it is now between the majority who have the qualifications for higher education and a minority whose qualifications (if any) signal failure.

### Apprenticeships

In the 1980s, UK governments rejected ‘apprenticeship’ as a name for their successive versions of youth training. Apprenticeships were considered old fashioned and in decline which was the case numerically although not in prestige. Governments expected, or at least hoped, that ‘youth trainee’ would become a superior brand. This did not happen, traditional apprenticeships survived and in 1994 the government began to fund ‘modern apprenticeships’. Research quickly began to show that apprentice training boosted earnings to the same extent as university degrees, and indeed that the earnings of ex-apprentices overlapped with graduates’ salaries (Bratsberg et al. 2020; McIntosh 2007; Paton 2014; Sutton Trust 2014). ‘Modern’ was subsequently dropped from the title, and by 2015 the apprentice ‘brand’ was being applied to nearly all government-supported training. The main exceptions today are ‘traineeships’ which are for young people who leave education but are not considered apprentice ready. By 2018–2019 around a third of all young people in England were entering a government-supported apprenticeship. These are not targeted at the unemployed, NEETs or any other ‘at risk’ group. They are for all ages and at all levels from basic (called intermediate) to post-graduate (see Table 1). Apprenticeships are always employer-led. The apprentices are always employees and are counted as employed in Labour Force Surveys. If over 19 and beyond the first year of an apprenticeship, they are paid at least the legal minimum wages for their ages. Advanced and higher apprentices will always start on at least the minimum wage and often significantly more. Employers want to recruit ‘the best’ and they are competing with higher education. Apprenticeships for young people aged up to 24 can be from 1 year to 5 years in length. For older age groups, apprenticeships need be no longer than 6 months.

**Table 1:** Apprenticeship starts by age, August 2018–July 2019 (in thousands)

	< 19	19–24	Older	Total
Intermediate	49	36	46	131
Advanced	35	52	74	162
Higher	4	19	48	71
Total	89	107	168	364

Source: Department for Education

Apprenticeships are at three levels as follows: intermediate, advanced, and higher (which include degree apprenticeships). For 18/19 year olds who have the option of university, advanced and higher apprenticeships, or equivalent jobs, are the alternatives. Around a quarter of young people who could apply to and then enter a university opt for these career routes. They amount to around a sixth of the age group.

Advanced and higher apprenticeships feed into two clusters of occupations (employment classes). One is a new upper working class. This class is tiered. Top tier remuneration equals that of lower-level professionals and managers. The jobs are in construction and allied trades (electricians and plumbers, for example), aerospace, motor assembly, repairs and component manufacture, other manufacturing industries, sections of public transport such as railways, water management, distribution and waste disposal, telecommunications, energy supply and distribution, and other business sectors. This employment class accounts for around 15% of the present-day workforce (Wilson and Homenidou 2012). An advanced level apprenticeship will qualify an individual for craft work. Higher apprenticeships lead to higher technician grades, and degree apprenticeships feed into elite professional careers.

A second, larger cluster of occupations is a lower middle class who are employed in offices and laboratories, often in front of computer screens. An advanced apprenticeship will provide training in basic office skills. A higher apprenticeship will feed into a career in accountancy, marketing, human resource management, and other non-manual specialisms. Contrary to forecasts of an hour-glass shaped occupational structure resulting from a decline in skilled manual occupations (which has happened) and intelligent computers replacing office staff, the latter grades have expanded as the new information and communication technologies have enabled more work to be taken-on, and much lower-level professional and management work has been downgraded into the lower middle class. Jobs in this class account for around 45% of all employment (Wilson and Homenidou 2012).

Youth jobs that feed into these employment classes are not all government-supported apprenticeships. Many firms prefer to recruit and train young people outside the government programme. One reason is to avoid ‘red tape’. A second reason for not seeking government support is a desire to customize training to a firm’s specific requirements (Confederation of British Industry 2019; Dolphin and Lanning 2011; Field 2018; Fleckenstein and Lee 2018; McGurk and Meredith 2018). In Britain firms train for themselves, not for an industry or the economy in general. In 2017 the government introduced a training levy of 0.5% on all payrolls in excess of £3 million. Firms can recoup their contributions by training apprentices. This measure was intended to boost the quantity and quality of training. In the event, government-supported apprentice starts fell from 565,000 to 370,000 between the 12 months preceding and following the introduction of the levy (Richmond 2020). There was no corresponding decline in employment. Firms must have continued to recruit and train but without the greater bureaucracy and more prescriptive demands for receiving government support, and/or ‘fake’, cheap labour apprenticeships were culled. Since the post-levy drop, the number of apprentice starts per year has stabilized, and there has been a shift out of intermediate into higher apprenticeships. Approximately 110,000 young people in England started an advanced or higher government-supported apprenticeship in 2018–2019, and another 50,000 are likely to have entered equivalent jobs outside the government programme. Any young person with the option of higher education is unlikely to opt for an inferior apprenticeship or job. For 18/19 year olds, the big problem with advanced and higher apprenticeships is that there are not enough. Over 40% of all apprenticeships for young people aged up to 24 are at intermediate level. Only 12% of the total are higher apprenticeships. Degree apprenticeships in particular are heavily oversubscribed. Competition for places is as intense as for Oxbridge (see Engeli and Turner 2019; McEwan 2019; Office for Students 2019; Powell 2019).

For some young people, university is their goal, sometimes a specific course at a specific university. For others, nowadays, university is their default option if they are unable to obtain suitable apprenticeships or other jobs. Up to the early-1970s, university graduates could be described as an elite (Kelsall et al. 1972). University students had survived several waves of selection. They had been among the minority who passed the 11-plus and attended grammar schools, where they were among those who performed sufficiently well in 16-plus exams to be encouraged to continue into sixth forms where they needed to gain sufficiently good grades at age 18 to be admitted to universities where demand for places exceeded supply. Employers could be confident that university graduates were the country’s smartest young people. This is no longer the case. University has become the normal end point for young people’s careers in education. Most attend non-selective comprehensive secondary schools, where most perform sufficiently well in the GCSE examinations at age 16 to enrol on courses that will qualify them for university entry. Most who qualify do proceed to university (around 40% of all 18/19 year olds). Going to university is simply normal. Employers may well feel that graduates are no longer exceptionally smart. Simultaneously the increases in student fees from £1 K per year to £3 K in 2006, then to £9 K in 2012, and the replacement of maintenance grants with loans resulting in graduates carrying around £50 K in student debt, has enabled employers to make a competitive offer to smart 18 year olds with impressive results in school examinations. These are steps and boundaries in England’s transition regime that are currently under negotiation in local education and labour markets.

### Employment Growth and the Precariat

England’s third regime change driver has been the almost uninterrupted expansion of employment since 1992. Forecasts of jobless growth and bidding farewell to work have been confounded (Aronowitz and DiFazio 1994; Dunkerley 1996; Forrester 1999; Jenkins and Sherman 1981; Rifkin 1994). So have forecasts that future growth would be mostly in high-skilled, high value added, high salary knowledge jobs (Employment Department 1993, 1995; Reich 1997). In practice the strongest employment growth in England over the last 30 years has been at the bottom, creating an employment class identified by Standing (2011) and appropriately called a precariat. This class is the most likely destination of the one-in-three young people for whom higher education does not become an option. Their most likely first footholds in the workforce will be intermediate apprenticeships, possibly preceded by traineeships, or simply minimum wage jobs. Low pay at the start of working life is customary but routes upwards have shrunk for the twenty-first century precariat (see Avram and Harkness 2018; Smith 2009). The ‘no jobs’ nightmare of the 1980s which might have led to the formation of an underclass (see Mann 1991; Murray 1990) has been replaced by a wealth of poor jobs (Maguire 2018; McDowell 2019; Shildrick et al. 2012). The jobs are precarious in various senses which usually include the pay making livelihoods precarious. Also, the jobs are likely to be temporary or in businesses that themselves are fragile. The jobs may be part-time, and in 2017, England had almost a million workers employed on zero-hours contracts (Office for National Statistics 2017). The precariat work in hospitality (hotels, bars, cafes, restaurants) and in retail shops and distribution warehouses, and this class also includes a new self-employed army of cleaners, gardeners, and staff who deliver on foot, by cycle, and motorized transport (Trade Union Congress 2013).

These types of employment have grown in all European countries, but in Britain, the growth of low-paid jobs has been amplified by the widespread use of Working Tax Credits (currently being assimilated into Universal Credit) to lift households just above the official poverty line. Young workers are rarely eligible for wage top-ups, just the low pay, and they will be pressured to take virtually any job by a harsh and punitive welfare system (Taylor 2017). This enables youth unemployment and NEET rates to be kept below the current European averages (see Table 2).

**Table 2:** Labour Force Survey, June 2018, seasonally adjusted (in percentages)

	In full-time education	Not in full-time education
16–17		
Employed	20	7
Unemployed	6	2
Inactive	61	5
Total	87	14
18–24		
Employed	11	52
Unemployed	2	6
Inactive	20	10
Total	33	68

Source: Office for National Statistics, Labour Force Survey

Around a fifth of the workforce in England belongs to households that depend partly on welfare (Hick and Lanau 2017). Median salaries for young workers are only slightly higher than legal minimum pay. The gap between the minimum and median widens with age, but even at its widest around half the workforce can be considered low paid (see Table 3). Britain’s working poor have been the hardest-hit households by the recession that followed the financial crisis of 2007–09 and subsequent government austerity policies (Bell 2020; Clark and Heath 2014).

**Table 3:** Age and minimum and median pay per hour, 2019

National minimum and living wage	£	Median pay	Full-time males £	Full-time females £
25 and over, living wage	8.21	25 and over	16.95	14.55
21–24	7.70			
18–20	6.15	18–21	8.42	8.09
16–17	4.35	16–17	4.93	4.32
Apprentices and trainees	3.90			

Source: Office for National Statistics. Department for Work and Pensions; Labour Force Survey

The precariat is the most likely destination of young people who leave education with ‘nothing’, meaning no qualifications that have any value in the labour market. Their initial minimum wage jobs are unlikely to be steps upwards. The more attractive jobs are filled by young people who follow other routes.

### Financialization, Elite Careers and the Graduate Labour Market

University is the main route into elite careers although this is not the destination of most university graduates. Elite careers are a product of the post-1980s financialization of Britain’s corporate economy (see Lapavitsas 2011). This process started with the deregulation of finance businesses in the 1980s which immediately led to spectacular high earnings in ‘City’ occupations. The 1980s was the decade during which income inequalities widened dramatically. Real average household incomes rose by 28%, but for the richest fifth, it was 64% (Bell 2020). The rewards in City careers have been shared with the sections of professions that serve finance (mainly law and accountancy) and the senior managements in public limited companies that deliver returns on investments. The outcome has been elite careers leading into an upper middle class that has pulled well ahead of other management and professional occupations.

All graduates can compete for entry to elite careers, but they are not on a level playing field. Firms offering these careers usually actively seek recruits from no more than four, five, or six universities. Around 20 universities in total are involved (High Fliers Research 2019). Oxford, Cambridge, and the top London universities feature most frequently (Boliver 2015; Wakeling and Savage 2015). Graduates in medicine, dentistry, and veterinary science are assured of elite careers. Graduates in economics and engineering have the next best chances, but whatever the subject studied (doctors, dentists, and vets excepted) and whatever the university, 5 years after graduation, most of the two middle quartiles of graduates are earning middling salaries (£20 K to £30 K per annum) (Belfield et al. 2018; Department for Education 2017; Office for National Statistics 2019). They may rise higher, a few soar much higher (see Britton et al. 2020), but most cannot rise much higher because there is insufficient room above. Graduates who enter elite careers can expect to start on salaries of £30 K or more. It helps to live in or to be able to move to London where elite careers are most plentiful,\ and to be seen by recruiters as someone who will fit in, which tends to favour the privately educated (Friedman and Laurison 2019; Friedman et al. 2015; Ingram and Allen 2019; Montacute and Cullinane 2018). It helps immensely if students complete paid or unpaid internships with potential employers (Holford 2017; Vasagar 2011). The first contracts of those who survive earlier sifts are likely to be temporary, and if rewarded with a permanent job, the graduates find that they have joined another pool within which they must compete for career advancement up professional and management ladders towards the top 10% of income tax payers (over £55 K per year), then into the top 5 % (£85 K), and onwards to the top 1 % (£160 K). Around 15% of the UK workforce, comprising less than a half of all university graduates, embark on elite careers, but most do not ascend beyond the lower rungs: the spires narrow as they rise (see Roberts 2020).

Other graduates may join a profession’s lower ranks as high street solicitors and accountants or enter non-elite professions such as pharmacy, opticians, school-teaching, social work, and housing management. The financial rewards in elite careers have pulled so far ahead that lesser professions are better treated as upper tiers of a lower middle class than part of a unitary class of managers and professionals. Most graduates join the same office, laboratory, and workshop occupations that recruit through advanced and higher apprenticeships (Felstead et al. 2018; Henseke et al. 2018). In the 1980s, some youth training schemes acted as waiting rooms, withdrawing trainees from the labour market before returning them to the same job queues from which they had been recruited (Roberts and Parsell 1992). In England’s reconstructed transition regime, it is universities that act as waiting rooms. They offer longer but far more comfortable waits than the training schemes of the 1980s.

## Discussion and Conclusions

Young people entered the former, and now enter the twenty-first century transition regime, divided into two groups by their achievements in education. The difference is that the better-qualified have become by far the larger of the two groups. The three main employment classes into which young people could exit from the former transition regime have become four as a result of a precariat separating from an upper working class. Also, the main divide above the working class is no longer beneath all managers and professionals but beneath an upper middle class.

### Five Major Routes

From the eight possible journeys from two entry points to four employment classes, a pathway from failure at age 16 into an elite career looks impossible. Also, graduates should not need to drop into the precariat, although some are still in low salary jobs 5 years after exiting university, and there is anecdotal evidence of graduates simply lengthening their hours of work in student jobs in bars and retail establishments. Up to now, graduates’ earnings have soared ahead of other groups during their thirties (Britton et al. 2020). This trend may, but will not necessarily continue in the future. That said, transitions from higher education into long-term precarious low-paid jobs are unlikely to be normalized, expected and unremarkable routes. Employers offering minimum wage jobs with no prospects of career progression are unlikely to target university graduates. Journeys from university into the upper working class look unlikely since this would involve a prolonged period of training and the acquisition of vocational qualifications that are considered sub- rather than post-graduate. Thus, despite considerable meandering, there are just five main transition routes, some broader than others.

As presented earlier, the five routes are as follows:

i. University into elite careers
ii. University into the lower middle class
iii. Exit education at 18 and enter the lower middle class, possibly via an apprenticeship
iv. Exit education at 18 and enter the upper working class, possibly via an apprenticeship
v. Fail in education and join the precariat

### Regime-Wide Scanning

A merit of scanning an entire transition regime is that it permits identification of all the routes that individuals might follow, those which are alternatives, and those which are inaccessible to certain groups of young people. Although not discussed here, scanning an entire transition regime also makes it possible to pinpoint the career steps and junctures where place, class, gender, ethnic, and other social divisions make a difference. For example, females are over-represented in the two-thirds who succeed at age 16, then all along the route that leads directly into then through higher education (see Arnot et al. 1996; Bursnall et al. 2019). Males are over-represented in the bottom third at age 16. Social class origins make a difference during every career stage and at every juncture, which is why it proves so difficult to change rates of relative inter-generational social mobility. If suppressed at one stage, class differences immediately appear elsewhere.

## Successful Reconstruction?

The regime in total may not look admirable, but the parts with which they engage must work adequately for many because the links between origins and destinations are constructed by the interactive agency of employers, training providers, managements of educational institutions, young people, and those who advise them. The regime runs with unemployment and NEET rates that are below the current European averages and delivers the fastest education-to-work transitions in Europe. In presenting labour market data, the European Union now defines youth as 20–34 year olds. In England, nearly all transitions are completed between ages 16 and 24. Nineteen is the average age when young people start their first full-time jobs (Armstrong 2019). This is not to say that by age 24 most young people are settled in jobs or even occupations that they will occupy long-term. It is rather that by age 24 processes of adult career development are taking over, and this occurs years before most young people in England have completed their family and housing life stage transitions.

The regime looks robust on account of its low rates of unemployment and NEET, and also because the links and boundaries between steps can be renegotiated in response to changes in the economy and labour market. The robust character of the regime may be regarded as a benefit, but it locks-in features that the country may regard as unwelcome problems. It locks-in a low productivity, low skill, low wage sector of the workforce and economy in which state income support throughout the greater part of the life course is necessary to keep the poor working. Pressured schools pressure their pupils. Those who struggle but gain adequate results at age 16 must continue with this desultory experience for at least another 2 years then maybe throughout higher education. Before long, parents who are still repaying their own student loans will be advising children on the latter’s next steps after secondary education.

During 2020, COVID-19 wreaked havoc throughout the transition regime. Teaching from primary to higher education moved online. Traditional examinations were replaced by grades awarded from combinations of mock exam results, course work, and teacher assessments. Homes became workplaces. Normal recruitment into all employment classes was suspended. On the ‘other side’, post-COVID-19, all may quickly return to the former normality or another reconstruction could be triggered. The transition regimes in every country will be subjected to a more severe stress test than anyone could have envisaged.
